# Whole pediatric liver graft in modified RAPID transplantation for unresectable colorectal liver metastases

**DOI:** 10.3389/ti.2026.17124

**Published:** 2026-09-10

**Authors:** Roberto Luca Meniconi, Nicola Guglielmo, Maria Lanzone, Francesca Mazzarotto, Marco Colasanti, Giammauro Berardi, Marco Angrisani, Germano Mariano, Davide Chiappori, Eleonora Garofalo, Giuseppe Maria Ettorre

**Affiliations:** Department of General Surgery and Organ Transplantation, San Camillo-Forlanini Hospital, Rome, Italy

**Keywords:** auxiliary orthotopic liver transplantation, colorectal liver metastases, modified RAPID transplantation, pediatric liver graft, transplant oncology

## Abstract

Liver transplantation has re-emerged as a therapeutic option for unresectable colorectal liver metastases (CRLM), but organ shortage and allocation concerns remain major limitations. The RAPID procedure combines auxiliary liver transplantation with delayed total hepatectomy, allowing small-graft use while the native liver provides temporary metabolic support. We report a modified RAPID transplantation, interpreted as a RAPID-derived staged auxiliary orthotopic liver transplantation (AOLT) strategy, using a whole pediatric graft in a 68-year-old woman with liver-only CRLM and sustained response to chemotherapy. The graft, from a 2-year-old donor (280 g; GRWR 0.4), had been declined by all pediatric centers due to non-standard infectious risk and was authorized by the Italian National Transplant Center within a regulated pathway. Favorable arterial anatomy preserved native right liver perfusion, while portal inflow management combined intentional surgical narrowing of the right portal vein with delayed portal vein embolization (PVE). At pre-PVE CT, graft volume was 350 cc (estimated GRWR 0.5); after PVE, it reached 580 cc (estimated GRWR 0.8), enabling completion hepatectomy at four weeks. At eight months, the patient had normal liver function and no recurrence. To our knowledge, this is the first reported use of a whole pediatric liver graft in modified RAPID transplantation.

## Graphical Abstract

**Figure:**
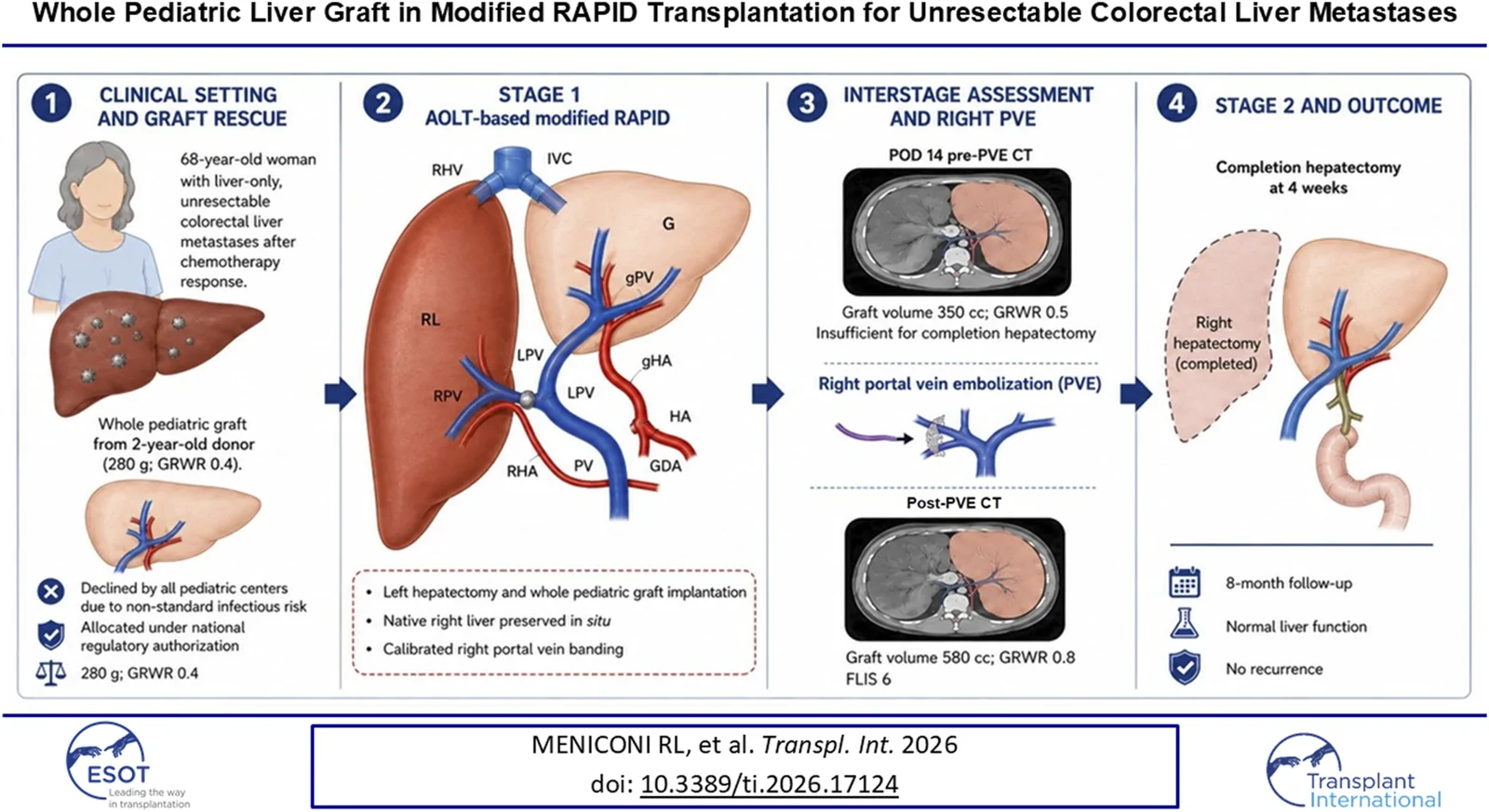

## Introduction

Liver transplantation (LT) for unresectable colorectal liver metastases (CRLM) has gained renewed interest after prospective experiences and the randomized TransMet trial demonstrated meaningful survival benefit in highly selected patients [1–4]. Nevertheless, the use of standard orthotopic LT for CRLM remains constrained by organ shortage, allocation ethics, and competition with established transplant indications. These limitations have stimulated increasing interest in transplant oncology strategies that may reduce the impact of CRLM transplantation on the conventional donor pool [5].

The RAPID (Resection and Partial Liver Transplantation with Delayed Total Hepatectomy) procedure was proposed to address some of these limitations by combining auxiliary liver transplantation with staged hepatectomy [6]. In the original description, a small partial graft, typically segments 2–3, is implanted during the first stage while the native liver provides temporary metabolic support during graft hypertrophy and functional recovery. Completion hepatectomy is subsequently performed once adequate graft volume and function have been achieved. International consensus recommendations have recently been published to standardize patient selection and procedural conduct [7].

Published RAPID experiences have generally relied on split left lateral segment grafts obtained from deceased or living adult donors. This approach is consistent with the original rationale of using small grafts and preserving conventional allocation opportunities [8, 9]. However, reduced graft volume may pose technical and functional challenges, particularly in adult recipients, making careful inflow modulation, graft monitoring, and timing of completion hepatectomy essential.

The use of a whole pediatric liver graft instead of a conventional split left lateral segment graft has not been previously described in the RAPID setting. Because the present case used a whole graft and a sequential portal inflow strategy rather than complete right portal vein ligation at stage 1, it should be interpreted as a RAPID-derived staged auxiliary orthotopic liver transplantation strategy (AOLT) rather than canonical RAPID sensu stricto. A fundamental prerequisite for any such use is that it must not compete with pediatric transplant candidates. We report a case in which a whole pediatric graft, formally declined by all pediatric transplant centers due to non-standard infectious risk, was allocated under national regulatory authorization within an approved transplant oncology protocol for unresectable CRLM. This report focuses on technical feasibility, perioperative assessment, graft regeneration, intentional surgical portal inflow management, and allocation considerations.

## Methods

The present study was conducted within the RAPID–Padova institutional protocol for unresectable CRLM, approved by the local ethics committee and registered at ClinicalTrials.gov (NCT04865471). Written informed consent was obtained from the patient for publication of the case and accompanying images.

Eligibility for transplantation was assessed by a multidisciplinary transplant oncology board. Selection criteria included unresectable liver-only CRLM, sustained response to systemic therapy, absence of extrahepatic progression, favorable tumor biology, and adequate performance status. Molecular tumor profile, radiological response, tumor marker trend, and prior resection of the primary tumor were all considered during evaluation. The availability of a rescue or otherwise non-utilized graft was not considered a reason to broaden oncological eligibility criteria.

Preoperative vascular anatomy was reviewed to assess feasibility of AOLT, preservation of native liver perfusion during the interstage period, and subsequent completion hepatectomy. Particular attention was paid to hepatic arterial anatomy, portal inflow configuration, venous outflow reconstruction, and biliary reconstruction options.

Donor graft allocation followed a stepwise process aligned with Italian national transplant regulations. The graft was formally offered to all pediatric transplant centers according to standard allocation priority. Only after exhaustion of all pediatric allocation options, and following assessment by an infectious disease specialist, was the graft considered for adult use within the modified RAPID strategy. Specific written authorization from the Italian National Transplant Center was obtained before graft acceptance. This authorization was protocol-specific and did not set a precedent for routine pediatric-to-adult graft allocation in oncological transplantation. Donor infectious risk, blood culture results, respiratory microbiology, antibiotic coverage, and overall donor risk assessment were reviewed and documented before acceptance.

Perioperative feasibility was assessed according to technical completion of stage-1 modified RAPID transplantation, graft-to-recipient weight ratio (GRWR), portal inflow modulation strategy, Doppler ultrasound assessment, liver function during the interstage period, radiological graft volume, gadoxetic-acid functional liver imaging score (FLIS; range 0–6), completion hepatectomy, postoperative liver function, and early oncological follow-up.

## Results

### Recipient and oncological selection

A 68-year-old woman with no major comorbidities was diagnosed with adenocarcinoma of the splenic flexure and synchronous bilobar CRLM. Molecular profiling demonstrated RAS and BRAF wild-type disease. Systemic treatment with FOLFOX plus panitumumab achieved sustained radiological response and normalization of tumor markers. Despite favorable tumor biology and treatment response, liver resection was not feasible because of the initial bilobar distribution and insufficient future liver remnant.

After resection of the primary tumor, restaging confirmed liver-only disease without extrahepatic progression. The patient had Eastern Cooperative Oncology Group (ECOG) performance status 0 and was accepted for transplantation within the institutional protocol. Standard orthotopic LT was not considered a realistic option given the patient’s oncological indication, the allocation priority framework applicable in Italy, and the absence of a suitable adult deceased donor split graft within a clinically acceptable timeframe.

A favorable vascular configuration was identified preoperatively, with a replaced right hepatic artery arising from the superior mesenteric artery and a common hepatic artery supplying the left liver. This anatomy was considered advantageous because it allowed preservation of arterial inflow to the native right liver during the interstage period while enabling direct arterial reconstruction of the graft at stage 1.

### Donor and graft allocation

A whole liver graft from a 2-year-old, 15-kg donor was considered for allocation. The donor had experienced post-anoxic encephalopathy after drowning and was classified as carrying non-standard infectious risk following infectious disease assessment, due to elevated inflammatory markers, bilateral pneumonia on imaging, and positive bronchoalveolar lavage cultures despite persistently negative blood cultures. Broad-spectrum antibiotic therapy with piperacillin/tazobactam had been initiated before procurement.

The graft was formally offered to all pediatric transplant centers in Italy in accordance with national allocation protocols. All contacted pediatric centers declined the graft, citing the non-standard infectious risk classification and its associated uncertainty in a pediatric recipient population. Only after exhaustion of all pediatric allocation options was the graft considered for use within the modified RAPID strategy. Organ utilization in the adult recipient was authorized by the Italian National Transplant Center within the approved protocol and did not require modification of standard antimicrobial prophylaxis for liver transplantation.

### Stage-1 modified RAPID transplantation

Stage-1 modified RAPID transplantation consisted of left hepatectomy extended to the middle hepatic vein and auxiliary orthotopic implantation of the whole pediatric graft. The measured back-table graft weight was 280 g, corresponding to a GRWR of 0.4. The graft was preserved using University of Wisconsin solution, and cold ischemia time was 480 min.

**FIGURE 1:**
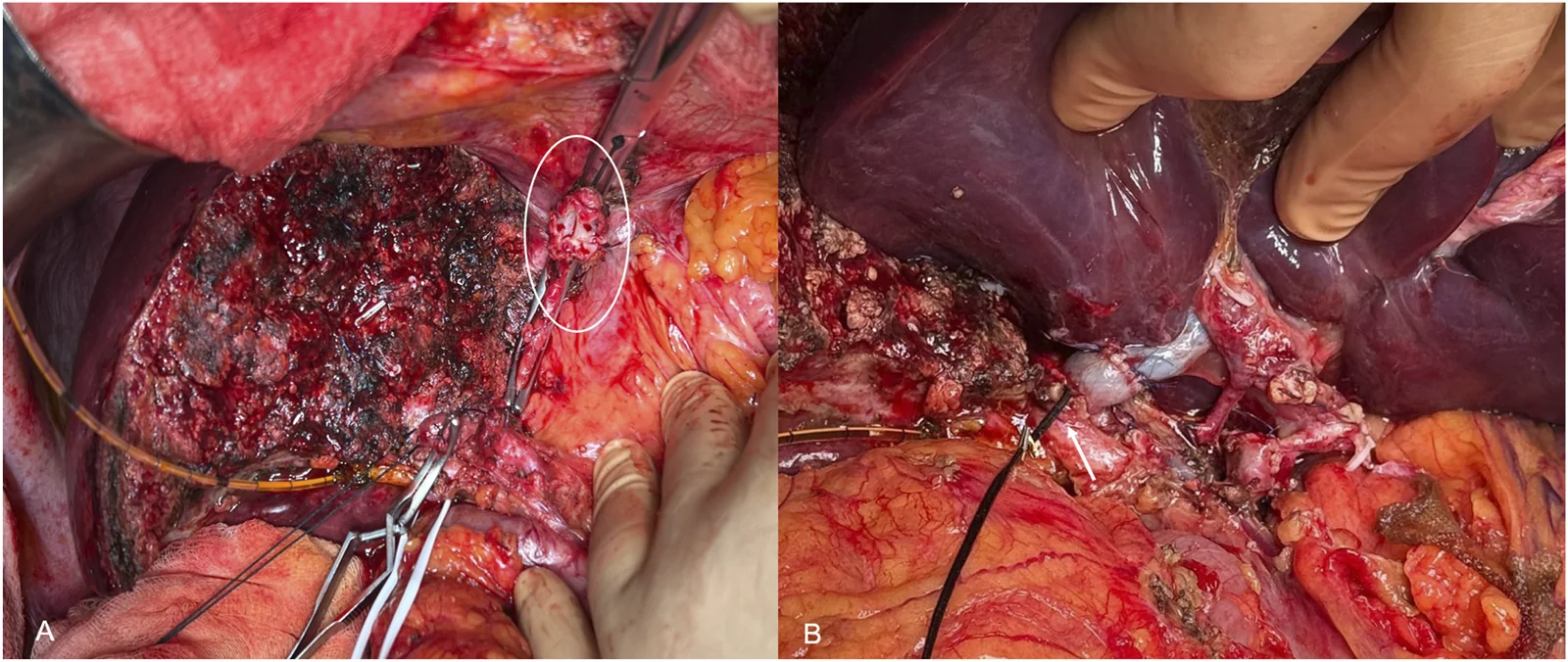
**(A)** Left hepatectomy extended to the middle hepatic vein; a venous cuff formed by the common trunk of the middle and left hepatic veins is shown above the clamp. **(B)** Whole pediatric liver graft implanted in an auxiliary orthotopic position. Arrow shows intentional surgical narrowing of the native right portal vein.

**FIGURE 2:**
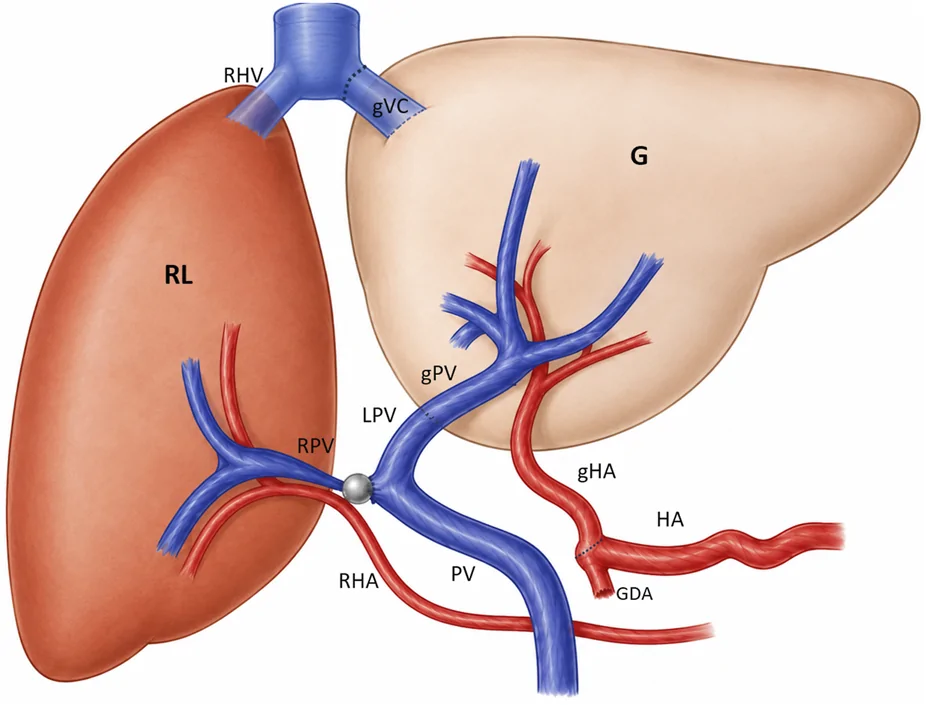
Schematic representation of stage-1 modified RAPID transplantation using a whole pediatric liver graft (G), within a RAPID-derived staged auxiliary orthotopic liver transplantation strategy. The native right liver (RL) is preserved with arterial inflow from a replaced right hepatic artery (RHA) arising from the superior mesenteric artery. The graft hepatic artery (gHA) is anastomosed to the recipient common hepatic artery (HA), the graft portal vein (gPV) to the native left portal branch (LPV), and venous outflow is reconstructed using the recipient common trunk of the middle and left hepatic veins and the graft vena cava (gVC). Portal inflow is managed by intentional surgical narrowing of the right portal vein. Biliary reconstruction is performed using Roux-en-Y hepaticojejunostomy (not shown).

Venous outflow reconstruction was performed using the recipient common trunk of the middle and left hepatic veins and the graft vena cava. The graft hepatic artery was anastomosed directly to the recipient common hepatic artery at the level of the gastroduodenal artery, while native right liver arterial inflow was preserved through the replaced right hepatic artery arising from the superior mesenteric artery. Portal anastomosis was performed between the native left portal branch and the graft portal vein. Portal inflow management was achieved by intentional surgical narrowing of the right portal vein rather than complete ligation, aiming to reduce abrupt hyperperfusion of the small pediatric graft while maintaining residual portal inflow to the native right liver. The degree of narrowing was guided by graft size, intraoperative macroscopic assessment of graft perfusion and congestion, and the need to preserve native right liver perfusion (Figures 1, 2). Biliary reconstruction was performed using Roux-en-Y hepaticojejunostomy. Doppler ultrasound confirmed patency of the portal and arterial anastomoses, hepatopetal portal flow, and preserved arterial signal. Quantitative portal flow and direct portal pressure measurements were not obtained.

### Interstage assessment and completion hepatectomy

**FIGURE 3:**
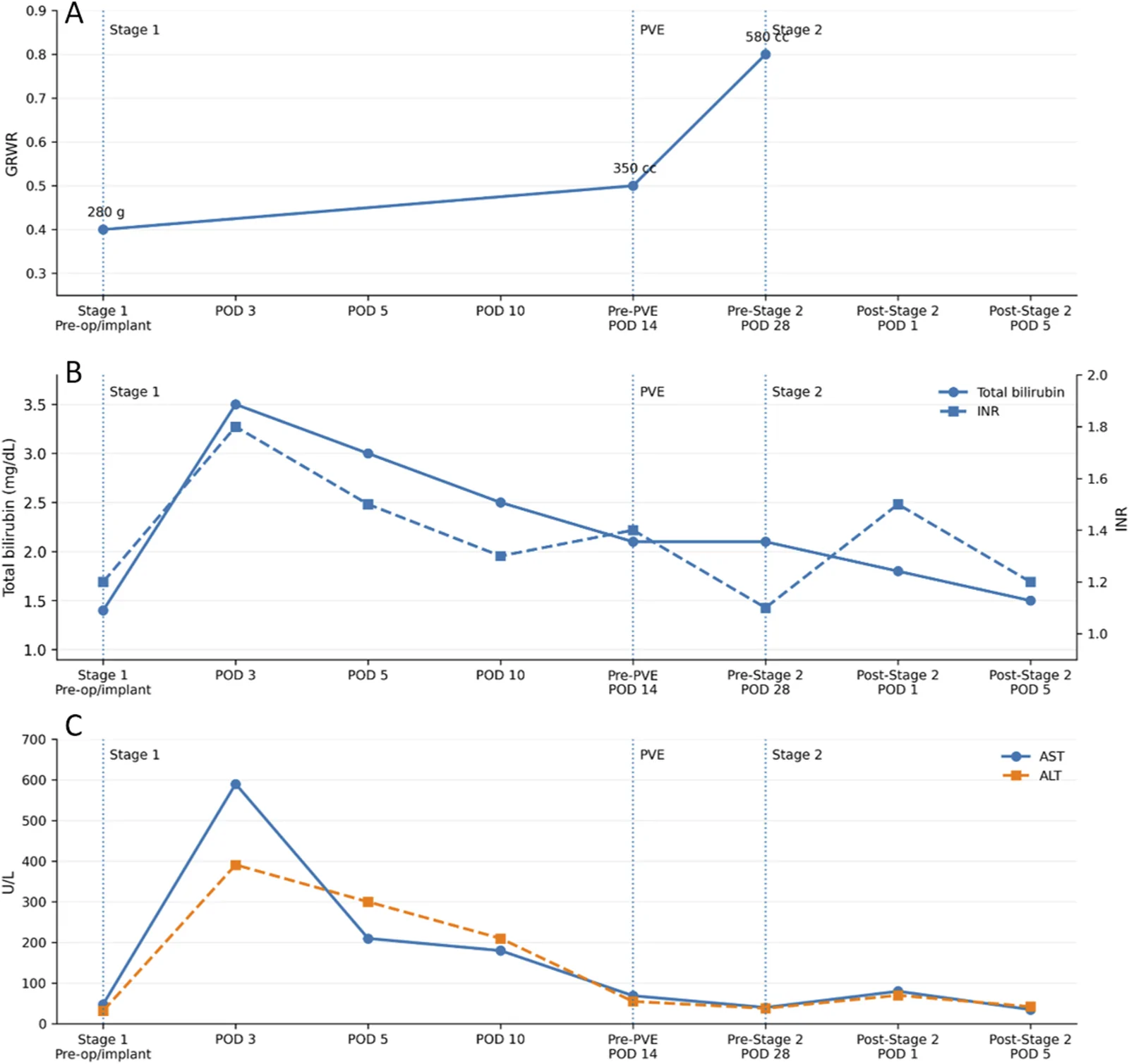
Volumetric and functional kinetics during modified RAPID transplantation. **(A)** Graft size and GRWR kinetics. **(B)** Total bilirubin and INR trend. **(C)** AST and ALT trend. PVE, portal vein embolization; GRWR, graft-to-recipient weight ratio; POD, post-operative day.

**FIGURE 4:**
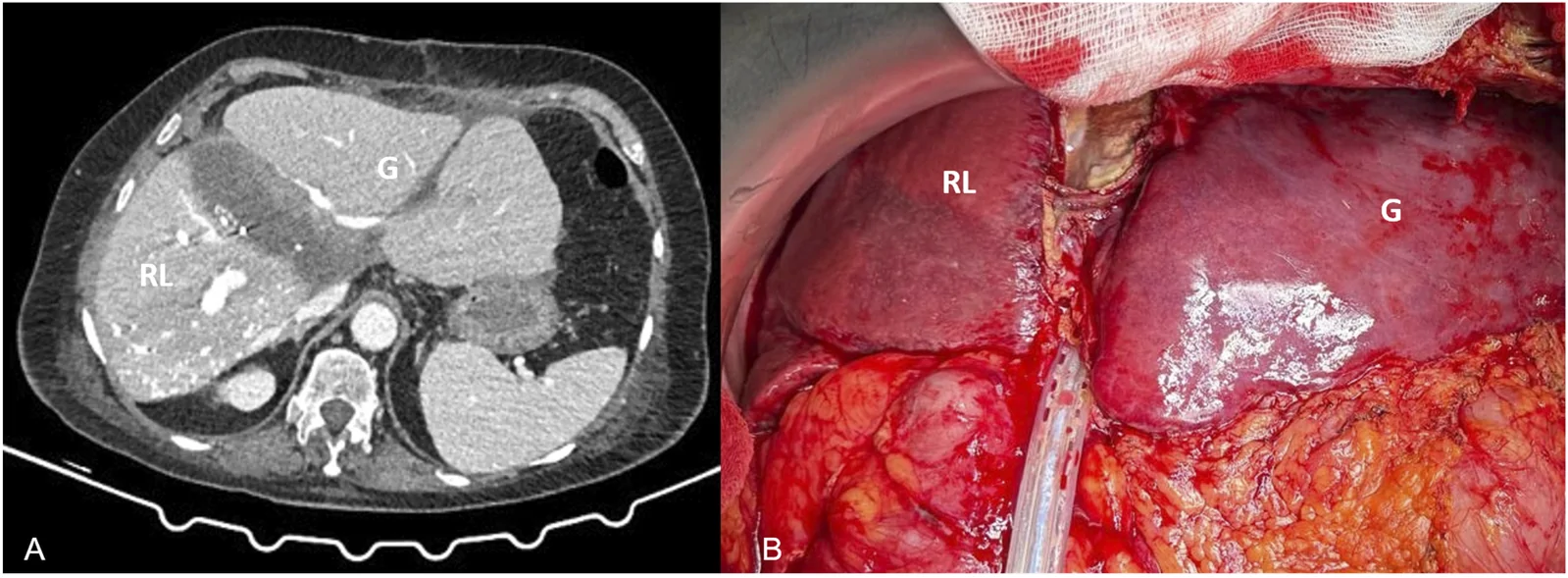
Perioperative imaging and intraoperative findings. **(A)** Postoperative contrast-enhanced CT scan after right portal vein embolization showing marked hypertrophy of the pediatric graft following stage-1 modified RAPID transplantation. **(B)** Intraoperative view of the second stage before completion right hepatectomy. RL, right native liver; G, pediatric graft.

Volumetric and functional kinetics during the interstage period and after completion hepatectomy are shown in Figure 3 and Supplementary Table S1. Recipient weight was approximately 70 kg. The measured back-table graft weight was 280 g, corresponding to a GRWR of 0.4. At the pre-PVE CT assessment, graft volume was 350 cc, corresponding to an estimated GRWR of 0.5. This was considered insufficient to proceed safely to completion hepatectomy. Therefore, right portal vein embolization was performed on POD 14 to further redirect portal inflow toward the graft and promote additional hypertrophy. Two weeks after PVE, graft volume had increased to 580 cc, corresponding to an estimated GRWR of 0.8. The post-PVE graft volume increase was 230 cc over 14 days, corresponding to 16.4 cc/day and a relative increase of approximately 65.7%. Liver function had also improved, with total bilirubin 2.1 mg/dL, AST 40 U/L, ALT 38 U/L, and INR 1.1. Hepatobiliary contrast-enhanced MRI confirmed preserved graft function, with a gadoxetic-acid functional liver imaging score (FLIS; range 0–6) of 6. Completion right hepatectomy was therefore performed 4 weeks after stage 1 (Figure 4).

No post-hepatectomy liver failure occurred. Ascites production was initially moderate and resolved progressively during the first postoperative week. At 8-month follow-up, the patient was alive with normal liver function and no radiological evidence of disease recurrence.

## Discussion

This report describes the technical feasibility of using a whole pediatric liver graft in a modified RAPID transplantation strategy for unresectable CRLM. Its primary ethical premise is that the graft used in this case was not in competition with any pediatric transplant candidate: it had been formally declined by all contacted pediatric transplant centers due to non-standard infectious risk, and its allocation to an adult recipient occurred only under specific regulatory authorization from the Italian National Transplant Center within an approved institutional protocol. This report should therefore be interpreted as documenting the rescue use of an otherwise non-utilized graft, not as a proposal for routine pediatric-to-adult allocation in transplant oncology.

The terminology of the present procedure deserves clarification. Canonical RAPID, as defined in the original description and in recent consensus recommendations, relies on partial orthotopic liver transplantation using a small graft, typically segments 2–3, followed by delayed total hepatectomy after adequate graft volume and function have been achieved [6]. The present case preserved the staged logic of RAPID, namely auxiliary orthotopic graft implantation, temporary native liver support, graft hypertrophy, and delayed completion hepatectomy. However, it differed from canonical RAPID in two key aspects: the use of a whole pediatric liver graft rather than a partial graft, and the adoption of sequential portal inflow management rather than complete right portal vein ligation at stage 1. For this reason, the present procedure is better described as a RAPID-derived staged AOLT strategy rather than canonical RAPID sensu stricto.

The portal inflow strategy also differed from canonical RAPID. In the original RAPID concept, complete right portal vein ligation is intended to aggressively redirect portal flow toward the graft and stimulate rapid hypertrophy. In the present case, intentional surgical narrowing of the right portal vein was initially performed to reduce the risk of abrupt hyperperfusion of a small pediatric graft in an adult recipient. Delayed right PVE was then used as a second step to further redirect portal inflow and stimulate graft hypertrophy. This sequence may have combined small-for-size protection with delayed hypertrophic stimulation, although the lack of direct portal pressure and quantitative flow measurements prevents precise assessment of the relative contribution of each maneuver.

The availability of intermediate volumetric and functional assessment helps clarify the rationale for sequential inflow management. At the pre-PVE CT assessment, graft volume was 350 cc and estimated GRWR was 0.5, which was considered insufficient for safe completion hepatectomy. Delayed right PVE was therefore performed as a second step. Before stage 2, graft volume had increased to 580 cc and estimated GRWR to 0.8, with preserved functional assessment. The post-PVE increase of 230 cc over 14 days, corresponding to 16.4 cc/day and approximately 65.7% relative growth, supported the decision to proceed to completion hepatectomy. This sequence suggests that the initial narrowing strategy may have protected the small pediatric graft from abrupt portal hyperperfusion, whereas delayed PVE provided the additional hypertrophic stimulus required for stage 2. However, because direct portal flow and pressure measurements were not obtained, the relative contribution of each maneuver cannot be precisely quantified.

The distinction between conventional LT for CRLM and modified RAPID transplantation is important. While the TransMet trial and prospective experiences support LT in selected patients with unresectable CRLM [3, 4], these data refer primarily to standard orthotopic transplantation [4]. RAPID and RAPID-derived strategies remain evolving approaches supported by limited clinical experience and recently published international consensus recommendations [7]. In this setting, objective documentation of graft volume and function before completion hepatectomy is essential, and the present case met both volumetric and functional criteria before proceeding to stage 2.

Several technical aspects merit discussion. First, favorable recipient arterial anatomy facilitated the procedure. The replaced right hepatic artery from the superior mesenteric artery allowed arterial inflow to the native liver remnant to be maintained throughout the interstage period, while the recipient common hepatic artery was available for direct graft reconstruction. However, this anatomical configuration should be considered advantageous rather than mandatory. In other settings, end-to-side anastomosis of the graft artery to the common hepatic artery may be feasible, with only temporary clamping and preservation of arterial inflow through the gastroduodenal artery or collateral pathways. Therefore, a replaced right hepatic artery should not be interpreted as an absolute prerequisite for modified RAPID transplantation, although it may simplify arterial management.

Second, the use of a whole pediatric graft differs technically from a split left lateral segment graft. The intact hilar and caval structure of the whole pediatric graft may simplify certain aspects of back-table preparation and implantation. At the same time, smaller vascular diameters, susceptibility to hyperperfusion injury, and the inherently limited functional reserve of a pediatric graft in an adult recipient require meticulous surgical planning and postoperative surveillance [9]. Doppler ultrasound confirmed vascular patency in the present case, but quantitative portal flow and portal pressure measurements were not available.

From an ethical standpoint, this case satisfies the conditions that would be required to justify pediatric-to-adult graft allocation in this specific context: the graft was unavailable to pediatric recipients through formal exhaustion of the allocation protocol; its use was authorized by the national regulatory institution within a prospectively registered protocol; the recipient met rigorous oncological and functional selection criteria; and the procedure was conducted within a multidisciplinary oversight framework. Importantly, graft selection and recipient eligibility must remain entirely decoupled. The availability of a rescue or otherwise non-utilized graft must not broaden oncological indications, weaken recipient selection, or justify expansion beyond accepted transplant-oncology criteria. These conditions are not routinely met and are unlikely to recur frequently, which underscores that this experience does not support policy changes in pediatric organ allocation.

The main limitations of this report are its single-case design, short oncological follow-up of 8 months, and absence of direct portal pressure and quantitative portal flow measurements. No conclusion regarding long-term oncological efficacy can be drawn from a single case with this follow-up duration. In addition, without direct hemodynamic measurements, it is not possible to determine whether immediate complete right portal vein ligation would have produced faster hypertrophy or allowed a shorter interstage interval. Publication bias inherent to case reports with favorable outcomes should also be acknowledged. Nevertheless, this case demonstrates the technical feasibility of whole pediatric graft rescue within a RAPID-derived staged AOLT strategy in one carefully selected patient under exceptional regulated allocation conditions. Future prospective registries should systematically collect hemodynamic, volumetric, functional, and oncological data to define the reproducibility and safety of this approach.

## Data Availability

The original contributions presented in the study are included in the article/Supplementary Material, further inquiries can be directed to the corresponding author.
