# Supplementary material for: Whole pediatric liver graft in modified RAPID transplantation for unresectable colorectal liver metastases
Source: Transpl Int. 2026 Sep 10;39:17124. doi: 10.3389/ti.2026.17124 (PMC13601019; doi:10.3389/ti.2026.17124)
Supplement: Supplementary file 1 [file Table1.docx]

**Supplementary Table S1. Volumetric and functional assessment during modified RAPID transplantation.**

| **Timepoint** | **Graft size assessment** | **GRWR** | **Total bilirubin, mg/dL** | **AST, U/L** | **ALT, U/L** | **INR** |
| --- | --- | --- | --- | --- | --- | --- |
| Pre-stage 1 / implantation | 280 g, measured graft weight | 0.4 | 1.4 | 48 | 32 | 1.2 |
| POD 3 after Stage 1 | — | — | 3.5 | 590 | 391 | 1.8 |
| POD 5 after Stage 1 | — | — | 3.0 | 210 | 300 | 1.5 |
| POD 10 after Stage 1 | — | — | 2.5 | 180 | 210 | 1.3 |
| Pre-PVE, POD 14 | 350 cc, CT-derived graft volume | 0.5 | 2.1 | 69 | 55 | 1.4 |
| Pre-stage 2, POD 28 | 580 cc, imaging-derived graft volume | 0.8 | 2.1 | 40 | 38 | 1.1 |
| POD 1 after Stage 2 | — | — | 1.8 | 80 | 70 | 1.5 |
| POD 5 after Stage 2 | — | — | 1.5 | 35 | 42 | 1.2 |

*Abbreviations: GRWR, graft-to-recipient weight ratio; PVE, portal vein embolization; POD, postoperative day; AST, aspartate aminotransferase; ALT, alanine aminotransferase; INR, international normalized ratio.*

*Note: The implanted graft size refers to measured back-table graft weight. Subsequent graft-size assessments refer to CT/MRI-derived graft volumes.*
